# Analyzing the Effect of Electrolyte Quantity on the Aging of Lithium‐Ion Batteries

**DOI:** 10.1002/advs.202405897

**Published:** 2024-08-19

**Authors:** Christian‐Timo Lechtenfeld, Julius Buchmann, Jan Hagemeister, Marlena M. Bela, Stefan van Wickeren, Sandro Stock, Rüdiger Daub, Simon Wiemers‐Meyer, Martin Winter, Sascha Nowak

**Affiliations:** ^1^ Münster Electrochemical Energy Technology (MEET) University of Münster Corrensstraße 46 48149 Münster Germany; ^2^ School of Engineering and Design Institute for Machine Tools and Industrial Management (iwb) Technical University of Munich (TUM) Boltzmannstraße 15 85748 Garching Germany; ^3^ Fraunhofer Institute for Casting, Composite and Processing Technology IGCV Am Technologiezentrum 10 86159 Augsburg Germany; ^4^ Helmholtz‐Institute Münster (HIMS) IEK‐12 Forschungszentrum Jülich Corrensstraße 46 48149 Münster Germany

**Keywords:** aging mechanisms, electrolyte characterization, electrolyte quantity, lithium‐ion batteries, *post‐mortem* analysis

## Abstract

Despite a substantial impact on various economic and cell technology factors, the influence of electrolyte quantities is rarely addressed in research. This study examines the impact of varying electrolyte quantities on cell performance and aging processes using three different electrolytes: LP57 (1 M LiPF6 in ethylene carbonate:ethyl methyl carbonate (EC:EMC 3:7 w/w), LP572 (LP57+2 wt.% vinylene carbonate (VC)) and LP57 + absVC (18.351 mg VC). Comprehensive analytical post mortem investigations revealed that continuous excessive electrolyte decomposition determines the performance of cells using LP57, leading to enhanced irreversible lithium‐ion loss and interphase thickening with increasing electrolyte volume. Impedance rise due to the growth of the interphase was also identified as the cause of degrading cell performance with rising amounts of LP572, attributed to an increasingly pronounced consumption of VC rather than electrolyte aging effects. By varying the electrolyte quantity while maintaining a constant amount VC within the cell system, the differences in cell performance were minimized, and observed deteriorating effects were suppressed. This study demonstrates the sensitive interdependence of electrolyte volume and additive concentration, practically affecting aging behavior. Comprehensively understanding the characteristics of each individual electrolyte component and tailoring the electrolytes to cell‐specific cell properties proves to be crucial to optimize cell performance.

## Graphical abstract

Tailoring electrolyte scales, such as electrolyte quantity and additive concentrations, to cell properties is considered beneficial, not only for improving cell performance and aging behavior but also for enabling better comparability of different cell studies. Accordingly, the tests and analytical techniques applied in this study can be considered a blueprint to optimize and evaluate the desired electrolyte formulations with respect to the cell design and material system used.

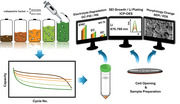

## 1 Introduction

A growing world population and the associated increase in industrialization as well as mobility leads to a globally rising demand for energy storage systems.^[^ ^1^ ^]^ In view of climate change, the electrification of the mobility sector is considered a key strategy to address the challenge of reducing global CO_2_ emissions. The lithium‐ion battery (LIB) has become a core technology for energy storage systems since its commercialization in 1991^[^ ^2^ ^]^ that is expected to continue growing exponentially in the upcoming years.^[^ ^3^ ^]^ To meet expectations for electric mobility applications, LIBs continuously require improvements, e.g., increased energy densities^[^ ^4^ ^]^ as well as a decrease in production costs.^[^ ^5^ ^]^

The production of LIBs consists of a series of production steps, each with individual challenges and impacts on cell quality.^[^ ^6^ ^]^ One of the last steps is the electrolyte filling process, in which a liquid electrolyte is brought into the cell stack consisting of anode, cathode, and separator. The electrolyte plays a decisive role by enabling the transport of lithium‐ions between the anode and cathode during the charging and discharging cycles.^[^ ^7^ ^]^ In order to ensure sufficient ionic conductivity, the porous structures of the electrodes and separator must be completely penetrated by the electrolyte. This wetting process can take hours or even several days, leading to a bottleneck in process time and a substantial cost factor in battery production.^[^ ^8^ ^]^ Experimental studies have shown that a wetting degree of at least 98 % is required before beginning cell formation to avoid lithium plating.^[^ ^9^ ^]^ Once the wetting is complete, the cell is charged for the first time, which is known as the formation process.

During formation, electrolyte components, such as the carbonate solvents and the conductive salt, are decomposed at the surface of the negative electrode. The organic and inorganic reaction products form an electronically insulating and lithium cation permeable layer on the surface of the negative electrode known as the solid electrolyte interphase (SEI).^[^ ^10^, ^11^ ^]^ Depending on the material system, the cell undergoes an irreversible capacity loss of ≈5–10 % during this first electrochemical step.^[^ ^12^ ^]^ Nevertheless, this procedure is considered essential as the formed SEI aims to suppress further degradation of the electrolyte, thereby mitigating capacity loss.^[^ ^10^, ^13^, ^14^ ^]^

However, the SEI cannot completely prevent electrolyte decomposition due to the lack of the properties of a true solid electrolyte, only allowing the transport of lithium‐ions, as well as the occurrence of phenomena such as volume expansion and cracking of active material during cell operation.^[^ ^10^, ^15^ ^]^ As a consequence, the electrolyte is continuously consumed throughout the cycle life, leading to a growth of the SEI and ultimately posing the risk of the cell drying‐out.^[^ ^10^, ^16^, ^17^, ^18^, ^19^ ^]^ To prevent drying‐out over the life span of the cell, a surplus of the required electrolyte quantity is used. It is important to consider, that any additional electrolyte that is not consumed during cycling adds to the cost and weight of the cell, ultimately decreasing gravimetric energy density.^[^ ^16^ ^]^ In contrast, film‐forming additives such as VC present one of the most economical and effective ways to improve the passivating characteristics of the SEI, reduce electrolyte decomposition, and prolong cycle life.^[^ ^20^ ^]^ However, the amount of additive requires sensitive dosing as different amounts are reported to cause unwanted performance impairments, such as the oxidative degradation of VC.^[^ ^21^, ^22^ ^]^ Thus, it is critical for cell manufacturers to optimize electrolyte quantity and composition.

The electrolyte quantity can be described as a volumetric factor (vf), which represents the ratio of electrolyte volume to the accumulated pore volume of anode, cathode, and separator. Nevertheless, the best‐performing vf is not uniformly defined, as it can vary depending on the cell design and material system used. Studies for different types of Lithium‐Nickel‐Manganese‐Cobalt‐Oxides (NMC) have shown that the ideal vf ranges between 1.4^[^ ^16^ ^]^ and 1.9^[^ ^23^ ^]^ based on the ratio of nickel, manganese, and cobalt. A change of anode material from graphite to silicon‐based was also demonstrated to affect the required vf.^[^ ^24^ ^]^ Previous studies in this field have focused on testing cell performance and the effect on the energy density. However, the underlying mechanism behind the electrolyte filling volume's impact on the performance is not yet well understood. Studies on the correlation of aging phenomena with changing vf are underrepresented in literature.^[^ ^16^, ^23^, ^24^, ^25^, ^26^ ^]^ Therefore, this study focused on the application of analytical techniques to investigate occurring aging processes of the cell system as a function of the electrolyte quantity and composition.

## 2 Experimental Section

### 2.1 Large‐Format Pouch Cells

#### 2.1.1 Cell Production

Large‐format pouch NMC622||graphite (G) cells with a nominal capacity of 5 Ah, produced at the Institute for Machine Tools and Industrial Management (*iwb*) of the Technical University of Munich (TUM), were used for electrochemical long‐term cycling investigations. All production steps from mixing to cell testing were performed in‐house on semi‐automated machines, as detailed by Schreiner et al.^[^ ^27^ ^]^ The cells were assembled in a dry room with a dew point of less than −42 °C. The cells consisted of 13 anodes and 12 cathodes, with an anode balancing factor of 20% (N/P ratio of 1.2). The anode consisted of 94% Graphite (SMGA5, Hitachi, Japan), 2% carboxymethyl cellulose binder (CMC MAC500LC, Inabata, Japan), 3% Styrene–Butadiene Rubber (SBR, Zeon, Japan), and 1% carbon black (Super‐C65, Imerys, Switzerland). The cathode consisted of 95% LiNi_0.6_Co_0.2_Mn_0.2_O_2_ (HED NCM‐622 DT011, BASF, Germany), 2.25% carbon black (Super‐C65, Timical, Switzerland), 0.75% additional conductive additive (SFG6L, IMERYS, Switzerland), and 1.5% polyvinylidene difluoride (PVdF Solef 5130, Solvay, Belgium). The detailed anode and cathode specifications are provided in **Table** **1**. The single‐sided mass loading is based on the entire electrode, and not only the active material. The separator used is a polymer‐based tri‐layer with a porosity of 39% and a thickness of 25 µm (2325, Celgard, USA).

**Table 1:** Electrode specifications of the large‐format pouch cells used for the experiments.

Electrodes (large‐format pouch cell)	Anode	Cathode
Active material	Graphite	NCM622
Single‐sided areal mass loading per side in mg cm^−2^	9.2	17.3
Porosity in %	30.2	31.7
Coating thickness in µm	150	130
Foil thickness in µm	10 (Cu)	15 (Al)
Length in mm	104	101
Width in mm	76	73

Before the filling procedure, cells were dried under vacuum at 80 °C for >12 h. Eight cells were filled with LP572 electrolyte with increasing vfs ranging between 1.01 and 1.58 in increments of ≈0.1. The experimental electrolyte volumes and according vfs are provided in Table S1 (Supporting Information). Sealing was performed at 50 mbar with the vacuum chamber being flushed three times with nitrogen gas before sealing to remove residual dry room air. The filled cells were wetted for 2 h before starting the formation. Formation and subsequent cycling conditions for the life cycle test are listed in **Table** **2**.

**Table 2:** Formation and cycling conditions of the life cycle test for the NMC622||G large‐format pouch cells.

Procedure (large format‐pouch cell)	Protocol
Formation	3 x C/10 CCCV (charge), CC (discharge) 2.9–4.2 V
Cycling	1C CCCV (charge), CC (discharge); 2 check‐up steps after each 50 cycles at C/10 & C/2 2.9–4.2 V

### 2.2 Small‐Format Pouch Cells

#### 2.2.1 Cell Assembly

Small commercial multi‐layer wounded NMC622||artificial graphite (AG) pouch cells (Li‐FUN Technologies Ltd., Hunan, China) with a nominal capacity of 200 mAh were used for electrolyte‐volume‐dependent aging investigations. The cells were dried overnight at 80 °C under vacuum. Filling and cell assembly was carried out in a dry room with a dew point of less than −42 °C. The electrolyte amount was referenced to the total pore volume calculated from the measures of the cell components and their respective porosities. The determined cell component specifications are listed in Table S2 (Supporting Information). Cells were filled with vfs ranging between 1.0 and 1.8 in increments of 0.2. Three different electrolytes with varying VC contents were used for the experiments: LP572 (2 wt.% VC) (Solvionic, Toulouse, France), LP57 (without additive) (Solvionic, Toulouse, France), and LP57 with a constant absolute amount of VC (18.351 mg) (prepared in‐house) added to each cell, regardless of the vf. The experimental electrolyte volumes and additive amounts are listed in Table S3 (Supporting Information). Each cell configuration regarding vf and used electrolyte was built and analyzed by threefold determination. The cells were sealed under vacuum (85 %) using a pouch bag sealer GN‐HS300V (Gelon LIB Group, Dongguan, China). Subsequently, the cells were wetted for 10 h before starting the cycling. The cells were electrochemically aged for 300 cycles using a MACCOR cell testing system (Series 400, MACCOR, USA) at 20 °C. The formation and cycling procedures are listed in **Table** **3**.

**Table 3:** Formation and cycling conditions of the NMC622||AG small‐format pouch cells for *post mortem* aging investigations.

Procedure (small‐format pouch cell)	Protocol
Formation	3 x C/5 CCCV (charge), CC (discharge) 2.9–4.2 V
Cycling	300×1C CCCV (charge), CC (discharge) last discharge CCCV (→ 1/10C) 2.9–4.2 V

### 2.3 Analytical Methods

#### 2.3.1 Mercury Intrusion Porosimetry

The porosity of each cell component (anode, cathode, and separator) was measured using a Pascal 140‐440 porosimeter (Thermo Fisher Scientific, Waltham, USA). The applied pressure ranged from 0.01 to 400 MPa, allowing access to pore sizes of 110–0.0037 µm. The determination consisted of a low‐pressure (0.01–0.4 MPa) and a high‐pressure measurement (0.1–400 MPa). Fifteen punched electrode samples (Ø12 mm) and ≈200–300 mg of separator were used for the measurement. Each cell component was characterized by triplicate. Blank measurements of the respective current collector foil and an empty dilatometer for the separator were carried out beforehand. The porosity calculation was performed using the SOLID software (Solver of Intrusion Data, version 1.2.1).

#### 2.3.2 Cell Opening and Electrolyte Extraction

The small‐format pouch cells were opened after electrochemical aging in discharged state in a glove box (O_2_ ≤ 0.1 ppm/H_2_O < 2.0 ppm) under argon atmosphere. The cell housing was cut open and the cathode was separated from anode and separator. The electrolyte was extracted via centrifugation of anode and separator using a MEGA STAR 600R centrifuge (VWR International, PA, USA) applying 9000 rpm at 20 °C for 20 min. Electrolyte samples were stored at −18 °C for later analysis.

#### 2.3.3 Gas Chromatography‐Flame Ionization Detection

Volatile solvent compounds and aging products in the extracted electrolytes were quantitatively investigated by external calibration using gas chromatography and flame ionization detection (GC‐FID). The experiments were executed on a Nexis GC‐2030 (Shimadzu, Kyoto, Japan) equipped with a Restek Rxi−5 ms (30 m × 0.25 mm, 0.25 µm; Restek GmbH, Bad Homburg, Germany) diphenyl dimethyl polysiloxane (5%/95%) fused silica column. The method was adapted according to Terborg et al.^[^ ^28^ ^]^ and GC parameters are listed in Table S4 (Supporting Information).

#### 2.3.4 Gas Chromatography‐Mass Spectrometry

The identification of electrolyte components and decomposition products of the aged electrolytes was carried out using gas chromatography‐mass spectrometry (GC‐MS). The measurements were performed on a GCMS‐QP2010 Ultra (Shimadzu, Kyoto, Japan) with an assembled AOC‐5000 Plus autosampler and a nonpolar Restek Rxi−5 ms (30 m × 0.25 mm, 0.25 µm; Restek GmbH, Bad Homburg, Germany) diphenyl dimethyl polysiloxane (5%/95%) fused silica column. Further parameters were applied according to Grützke et al.^[^ ^29^ ^]^ and GC parameters are listed in Table S4 (Supporting Information).

#### 2.3.5 Inductively Coupled Plasma‐Optical Emission Spectrometry

Electrode samples were punched from the middle of the electrode stack and washed with dimethyl carbonate. The samples were dissolved using a microwave digestion system Multiwave 7000 (AntonPaar, Graz, Austria). Inductively coupled plasma‐optical emission spectrometry (ICP‐OES) measurements were performed using an ARCOS (Spectro Analytical Instruments GmbH, Kleve, Germany) equipped with a Scott spray chamber, a cross‐flow nebulizer, and a radial positioned plasma torch. The following emission lines were observed during analysis: Li (670.780 nm), Ni (221.648 nm, 231.604 nm, 232.003 nm), Co (228.616 nm, 237.862 nm, 238.892 nm), Mn (257.611 nm, 259.373 nm, 403.076 nm), and Al (176.641 nm, 394.401 nm, 396.152 nm). The method and further parameters were adapted from Vortmann et al.^[^ ^30^ ^]^ and Evertz et al.^[^ ^31^ ^]^

#### 2.3.6 Scanning Electron Microscopy and Energy‐Dispersive X‐Ray Spectroscopy

The surface morphology of negative electrode samples was investigated by scanning electron microscopy (SEM) employing a CrossBeam 550 working station (Carl‐Zeiss AG, Jena, Germany) equipped with a field emission gun at an acceleration voltage of 3.5 kV.

Determination of the elemental composition of selected spots on the electrode surface was performed by energy‐dispersive X‐ray spectroscopy (EDX) with an Ultim Extreme detector (Oxford Instruments, Abingdon, UK) and evaluated with the AZtech software (Oxford Instruments, Abingdon, UK). Further parameters were adapted from Bela et al.^[^ ^32^ ^]^

## 3 Results and Discussion

### 3.1 Long‐Term Cycling Investigations of Large Format Pouch Cells

**Figure 1:**
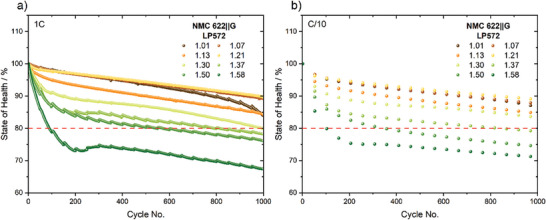
Normalized discharge capacities of NMC622||G large‐format pouch cells (5 Ah) with LP572 electrolyte at different vfs varying from 1.01 to 1.58. Life cycle test of 1000 cycles with one cell per vf was performed at 1C (a) and check‐up cycles for every 50th cycle at C/10 (b) in the voltage range of 2.9–4.2 V.

The influence of different electrolyte quantities on the electrochemical performance was investigated for large‐format pouch cells filled with varying vfs as described in Section 2.1. The cells were subjected to a life cycle test (Table 2) consisting of 1000 cycles at 1C with check‐up steps conducted after every 50th cycles at C/10 and C/2. **Figure** **1** shows the change in state of health (SOH) for the 1C life span cycles (a) and C/10 check‐up cycles (b) for each cell. The discharge capacities were normalized to the initial cycle at the corresponding C‐rate, respectively. The end of life (EOL) is usually defined as 80% SOH, highlighted by a horizontal line.^[^ ^33^, ^34^ ^]^

A decrease in the nominal capacity is evident for each vf and for both shown C‐rates. Overall, the cells with a lower vf of 1.07–1.21 exhibit a generally better cycling performance. This is particularly displayed by a moderate and linearly constant decrease in discharge capacity over the entire cycle life. However, in the case of vf 1.01, where the pore volume is filled with barely any excess, the cell performance is only stable until ≈600 cycles. At this point, an abrupt capacity fade termed as “roll‐over failure” or “sudden death” sets in triggered by a relatively advanced consumption of the electrolyte.^[^ ^17^, ^35^ ^]^ Due to this electrolyte degradation as well as immobilization, the cell becomes increasingly dry, decreasing the active regions of the electrode (loss of active material) and thus the cell capacity.^[^ ^17^, ^18^, ^19^ ^]^ Increasing the electrolyte volume to vf 1.21, not only increases the overall cell performance but also eliminates the strong degradation at the end of the life span, which is evident in the cell with vf 1.01.

Further increasing the electrolyte volume above vf 1.30 is not beneficial to cell performance as all cells reach the EOL criterion within 1000 cycles. The reason for the drawback of using higher electrolyte volumes is especially evident in the first 200 cycles, where an accelerated decrease in discharge capacity can be seen. The observed capacity drop becomes more pronounced with an increasing vf. Additionally, Figure 1b shows that the differently progressed capacity decay is independent of the C‐rate, since the overall trends of cell performance and vf are similar for C/10 and 1C. Based on the electrochemical data, an optimum for the electrolyte filling volume can be determined, resulting in the best possible cell performance for the respective cell system studied. This finding is consistent with previous investigations reported by Günther et al.^[^ ^16^ ^]^ and An et al.^[^ ^23^, ^24^ ^]^ Further analysis of the reasons leading to this phenomenon was attempted via *post mortem* analysis of the electrolyte and active materials. Due to the advanced aging process after more than 1000 cycles, a specific analytical examination proves to be difficult, as some cells were too dry for electrolyte recovery and electrodes accordingly too damaged for further analysis. In order to fully understand the responsible degradation phenomena, small‐format pouch cells were electrochemically aged and analyzed, as described in Section 2.2 Small‐Format Pouch Cells. The results are shown in Sections 3.2–3.5 Morphology Investigations.

### 3.2 Electrochemical Aging of Small‐Format Pouch Cells

Small‐format pouch cells were used to realize a defined electrochemical aging for subsequent *post mortem* analyses. Thus, the influences of aging processes responsible for the observed trends in cell performance during battery operation with varying electrolyte quantities can be identified. For this purpose, 300 cycles at 1C were chosen as operation criterium to not reach the cells’ end‐of‐life or provoke roll‐over failure. Additionally, the cycling and analytical *post mortem* investigations were performed on the three types of electrolytes to elucidate the influence of the electrolyte volume itself on cell performance and aging mechanisms, as well as varying amounts of film‐forming additive which is also affected by the electrolyte volume. This phenomenon was verbally discussed by Günter et al.^[^ ^16^ ^]^ and Klick et al.^[^ ^25^ ^]^ However, detailed experimental investigation separating the additive amount and the electrolyte volume are missing up to now.

**Figure 2:**
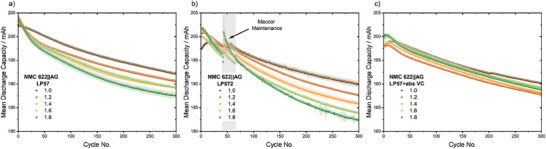
Discharge capacities of NMC622||AG small‐format pouch cells (200 mAh) with LP57 (a), LP572 (b) and LP57+absVC (c) electrolyte at different vfs varying from 1.0 to 1.8 (0.2 steps). Electrochemical cycling was conducted at 1C for 300 cycles in the voltage range of 2.9–4.2 V.

**Figure** **2** shows the mean discharge capacities of the small‐format pouch cells with varied electrolyte volumes between vf 1.0 and 1.8 (0.2 steps) of LP57 (a), LP572 (b) and LP57 with a constant absolute VC amount (18.351 mg) referred to as LP57+absVC (c).

Similar to the large‐format pouch cells with additive‐containing electrolytes (Figure 1a), the cells using LP57 without any additive (Figure 2a) also exhibit differences in electrochemical performance within the chosen 300 cycles. As previously observed for the long‐term cycling experiments, cell performance decreases with increasing vf. In terms of the selected test protocol, this leads to superior performance of cells with vf 1.0, as the electrolyte is not completely consumed during operation. Furthermore, compared to the large‐format pouch cells, the observed initial rapid capacity drop for larger electrolyte quantities is less pronounced, even with a higher vf of 1.8. However, the determined total pore volume does not take the dead volume of the cell into account, which can lead to distortions of the defined “real” vf.^[^ ^36^, ^37^ ^]^ Due to different dead volumes and in this case also different active materials, the required electrolyte volume and thus the cell performance differs between cell formats at the same vf.

In literature, the observed capacity fading during battery operation is mainly attributed to the reductive decomposition of the electrolyte components at the charged negative electrode, during which lithium‐ions are irreversibly immobilized.^[^ ^10^, ^14^, ^19^ ^]^ As previously introduced, the degradation products form the SEI on the electrode surface.^[^ ^10^, ^11^, ^13^ ^]^ The SEI formation and thus the greatest loss of lithium‐ions mainly takes place within the formation step, while the surface is still unprotected.^[^ ^19^, ^38^ ^]^ However, electrolyte decomposition proceeds at lower rates during prolonged cycling leading to a conversion and further loss of the electrolyte as well as growth of the SEI.^[^ ^10^, ^14^ ^]^ Considering the absence of additives, excessive electrolyte aging is a major factor for this irreversible capacity loss.^[^ ^39^ ^]^

Typically, film‐forming additives, such as VC, are used to modify the properties of the interphases (thickness, flexibility, organic/inorganic ratio, etc.) and to particularly inhibit the electrolyte aging processes, as these compounds reduce at a higher potential compared to EC.^[^ ^11^, ^40^, ^41^ ^]^ Overall, the cells using LP572 (Figure 2b) exhibit the same decreasing trend in electrochemical performance with increasing vf as already observed with LP57 electrolyte. However, a direct comparison of both electrolytes reveals that the difference between the individual vf is split to a greater extent using LP572, despite the utilization of VC. With increasing filling volume, this ultimately leads to increasingly poorer cell performance at the same vf. Since, as previously mentioned, the capacity fading is commonly associated with the continuous electrolyte degradation, this finding appears contradictory considering the actual positive properties of VC.^[^ ^41^ ^]^ As the reduction of VC also consumes lithium‐ions and leads to a change in cell impedance, the use of VC results in lower initial discharge capacities after cell formation compared to LP57.^[^ ^21^ ^]^ However, the normalized discharge capacities in Figure S1 (Supporting Information) show that between the vfs of 1.2 and 1.6 similar SOHs are achieved for the cells with LP57 and LP572 after 300 cycles. Merely at the vfs of 1.0 and 1.8, the utilization of VC leads to significantly improved and reduced SOH conditions, respectively. Thus, besides the pure electrolyte quantity, the total amount of additive added proves to have substantial influence on the cycling behavior as well. By using LP572 with a relative concentration of 2 wt.% VC, the total amount of additive within the cell system is increasing with the vf. Therefore, an excessive amount of the additive, which is not fully utilized during the formation cycles, can accordingly increase the irreversible capacity loss during further cycle life. Following the initial reduction process, VC can, among others, further form polymeric VC species, which extend on the electrode surface in the form of an effective organic SEI.^[^ ^41^, ^42^ ^]^ Thus, an excess of VC can also lead to thicker interphases, increasing the charge‐transfer resistance and limiting lithium intercalation kinetics.^[^ ^43^, ^44^ ^]^ As a consequence, this can result in plating of metallic lithium, which can subsequently react with the electrolyte to further accelerate cell aging.^[^ ^10^, ^45^ ^]^

For a more detailed analysis regarding the influence of the additive content on cycle life, cells were investigated in which the electrolyte quantity was varied while the absolute amount of VC was kept constant within the cell system. Figure 2c shows a diminishing difference in cell performance among the vfs using LP57 + absVC, eliminating the previously observed trend. These results suggest that the assumed increasing electrolyte degradation with increasing vfs can be minimized by exploiting the protective properties of VC, while negative effects caused by considerable excess of film forming agents can be excluded. The balance between the additive concentration and the electrolyte quantity, which is adjusted to the cell properties, accordingly plays an important role to optimize the cell system. However, if the mean discharge capacities are normalized to the initial cycle, the decreasing trend in SOH with increasing vf is still evident (Figure S1, Supporting Information). This is also noticeable in Figure 2c, as the cells with a higher vf possess a higher initial capacity in the first cycle after formation. A comparison of all three electrolytes reveals that higher electrolyte quantities generally provide higher initial discharge capacities. Despite having the best capacity retention over the life cycle span, the cells with vf 1.0 in particular exhibit the lowest initial values in all cases. However, the discharge capacities of these cells exceed the value of the initial cycle within the first 5–25 cycles for all tested electrolytes instead. While this is also partially present for cells at other vfs using VC, it is most pronounced for cells with vf 1.0. It is assumed that both observations are related to each other. The continuous recovery of the capacity during the early cycles can be ascribed to the anode overhang effect.^[^ ^46^, ^47^ ^]^ During the charging step of the cell formation at low C‐rates, the overhang area of the oversized anode is partly lithiated.^[^ ^46^ ^]^ Due to the formed interphases, but especially due to an insufficient wetting of the electrodes, transport of active lithium from the overhang area is kinetically hindered, resulting in the observed difference in usable initial capacity for cells with vf 1.0. However, the kinetic impairments lead to a slow recovery of the capacities in subsequent cycles as the active lithium is slowly fed back into the cell system.^[^ ^46^ ^]^

**Figure 3:**
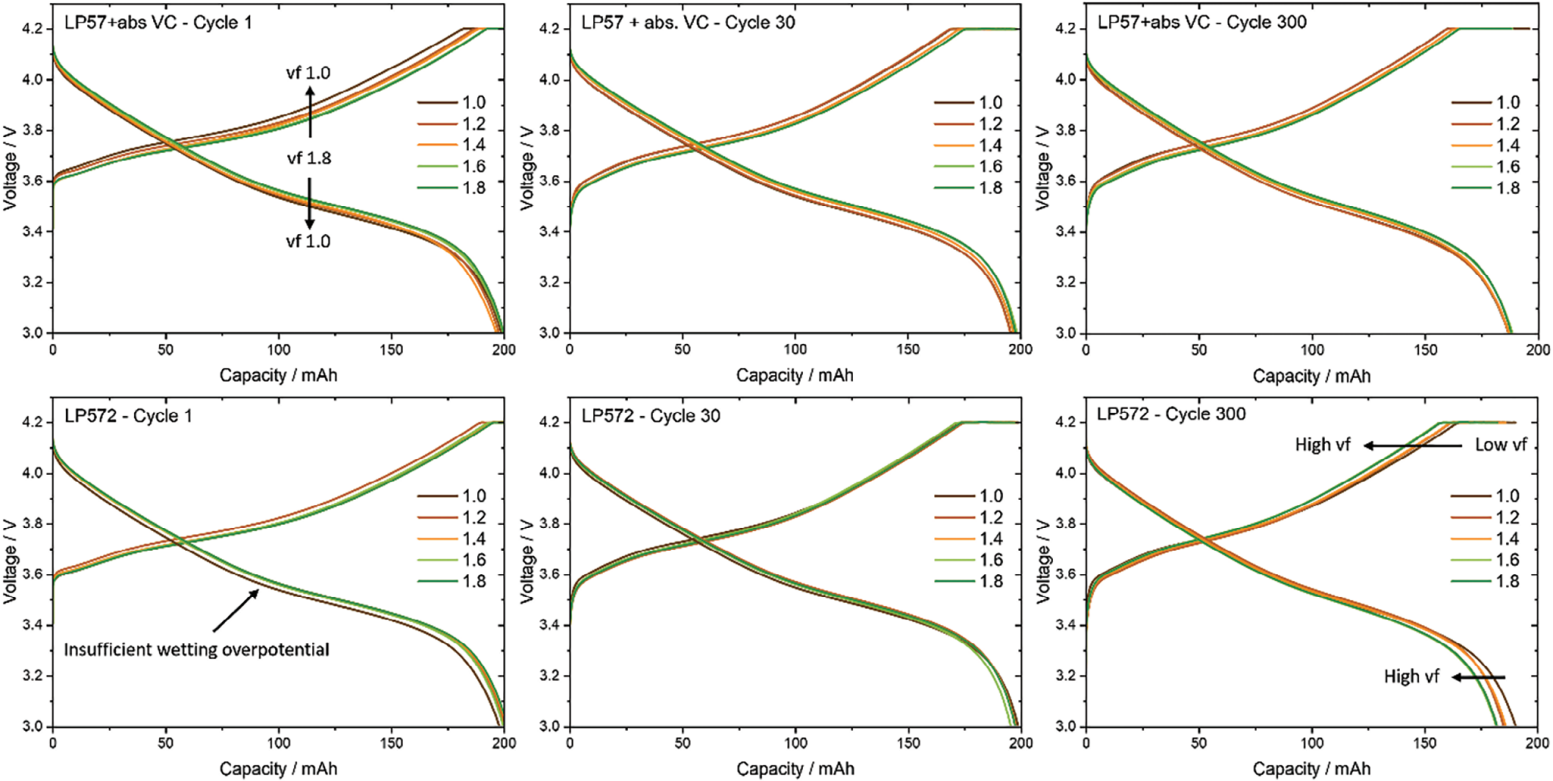
Charge and discharge curves at cycle 1, 30, and 300 after formation of the NMC622||AG small‐format pouch cells (200 mAh) with LP57 + absVC (top line) and LP572 (bottom line).

**Figure** **3** shows the charge and discharge curves of cells using LP57 + absVC and LP572 for the 1st, 30th, and 300th cycles.

During the first cycle, the charge curves of the cells using LP57 + absVC indicate a meaningfully higher overpotential at vf 1.0, whereby the cut‐off voltage is reached more quickly. In case of the remaining vfs, the overpotentials continue to decrease with increasing electrolyte quantities. The same trend is also observed within the discharge curves, where the ohmic drop for the cell vf 1.0 appears to be the largest, induced by a higher cell resistance. Since the absolute amount of VC inside the cell system is the same for all vfs, the difference within the overpotential should mainly be caused by the lack of electrolyte and thus an insufficient wetting for low vfs. Due to a progressive electrolyte distribution at low vfs as well as continuous consumption at higher vfs over time, the respective (dis)charge curves converge after 30 and 300 cycles. However, the observed overall trend in overpotentials remains the same.

For cells using LP572, the lack in electrolyte also appears to dominate in the first cycle. Solely the cells with low vf of 1.0 and 1.2 differentiate from the others due to a higher overpotential, which is presumably caused by insufficient wetting. While the differences disappear after 30 cycles as all (dis)charge curves largely overlap, the previously observed trend is reversed with cycle 300. Thus, the substantially increasing overpotentials with vf during cycling correlate with the rising excess of VC in the electrolyte and could be associated with a continuous growth of the interphases, potentially resulting in increasing cell impedances. While these findings reinforce the conclusions drawn from the life cycle tests, the cells were subsequently opened to further investigate the actual aging effects on the cells, focussing on the analysis of the electrolyte and active materials.

### 3.3 Electrolyte Analysis

**Figure 4:**
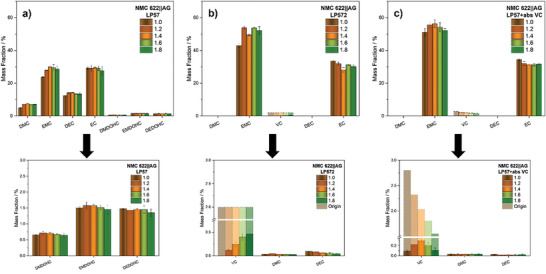
Mass fractions of volatile electrolyte components of the NMC622||AG small‐format pouch cells (200 mAh) with LP57 (a), LP572 (b), and LP57+absVC (c) at various vfs (1.0–1.8) after electrochemical aging. Low‐concentrated components are displayed enlarged with original VC mass fractions additionally presented transparently. Quantification was performed by means of GC‐FID.

In order to analyze the composition of the electrolytes after electrochemical aging, volatile electrolyte components and formed aging products were first identified via GC‐MS (Figure S2, Supporting Information) and subsequently quantified by means of GC‐FID. **Figure** **4** shows the determined mass fractions of the detected components for the different electrolytes, with low concentrated compounds additionally depicted enlarged below.

**Figure 5:**
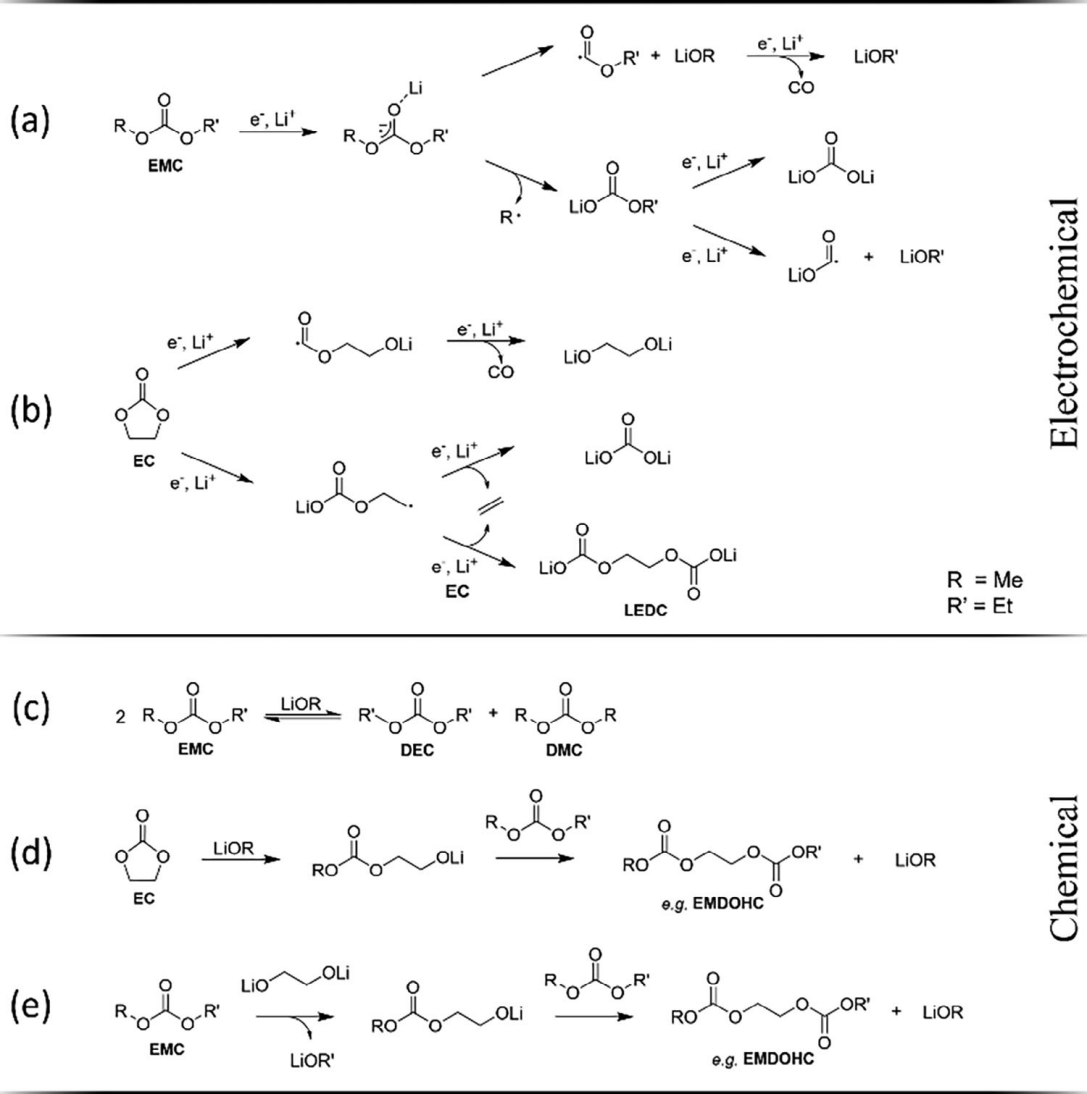
Reaction scheme for the electrochemical reduction pathways of EMC^[^ ^48^, ^50^ ^]^ and EC.^[^ ^48^, ^51^, ^52^ ^]^ Proposed chemical reactions of alkoxide triggered EMC transesterification^[^ ^50^, ^51^ ^]^ (c) and alkyl dicarbonate formation via EC^[^ ^48^, ^51^ ^]^ (d) and EMC^[^ ^57^ ^]^ (e).

While the baseline electrolyte LP57 is based on a mixture of EC and EMC in a ratio of 3:7, an explicit decrease in the mass fraction of the linear carbonate (originally ≈60 wt.%) for all vfs is noticeable after electrochemical aging (Figure 4a). The degradation due to electrochemical reduction of EMC, which is accompanied by irreversible immobilization of lithium‐ions, leads to the formation of inorganic lithium salts such as lithium carbonate, lithium alkyl carbonates and lithium alkoxides (**Figure** **5**a).^[^ ^48^, ^49^ ^]^ The latter was found to subsequently initiate the continuous degradation process of the electrolyte during prolonged cycling.^[^ ^48^ ^]^ In case of EMC, a nucleophilic attack of the alkoxide species on the carbonyl carbon induces a reversible transesterification to the linear carbonates DMC and DEC (Figure 5c).^[^ ^50^, ^51^, ^52^ ^]^ The presence of both conversion products of 5–10 wt.% further proves the advanced degradation of the linear carbonate in the aged electrolyte. According to Stockhausen et al.,^[^ ^53^ ^]^ however, the electrochemical reduction of the linear carbonate primarily takes place during the later phase of long‐term cycling, in which the electrolyte degradation is mainly driven by the abundance of the components. During formation and early cycles (≈25 cycles), especially in cells with additive‐free electrolytes, EC is preferentially decomposed, which is primarily associated with the initial SEI build‐up of the unprotected anode surface.^[^ ^54^ ^]^ However, a decrease in the EC mass fraction compared to the fresh electrolyte (≈26 wt.%) is not evident for any of the cells using LP57. Instead, the actual portions of 27–29 wt.% are slightly above the initial value. This can presumably be attributed to the concentration of the high boiling EC due to the evaporation of EMC during electrolyte extraction and/or stronger immobilization of the linear carbonate during cycling.^[^ ^55^ ^]^ Similar to EMC, the electrochemical reduction of EC, including the consumption of active lithium, leads to the formation of lithium salts, favoring the conversion to lithium ethylene dicarbonate (LEDC) alongside lithium carbonate and additional lithium alkoxide species (Figure 5b).^[^ ^48^, ^56^ ^]^ The lithium alkoxide species resulting from the reductive decomposition of EC/EMC provoke, among other products, the formation of alkyl dicarbonates, similar to the aforementioned transesterification (Figure 5d,e).^[^ ^48^, ^57^ ^]^ The presence of the three alkyl dicarbonates, dimethyl‐2,5‐dioxahexane carboxylate (DMDOHC), ethylmethyl‐2,5‐dioxahexane carboxylate (EMDOHC), and diethyl 2,5‐dioxahexane carboxylate (DEDOHC), is often used to assess the electrolyte health and evaluate the effectiveness of film‐forming additives.^[^ ^58^, ^59^ ^]^ These compounds are known to increase the cell impedance and thus deteriorate the cell performance. The higher viscosity of the alkyl dicarbonates affects the bulk properties of the electrolyte by lowering the ionic conductivity. Moreover, it was found that the surface film resistance is also considerably increased by the presence of these compounds, resulting in a likewise increased polarization of the graphite anode.^[^ ^57^, ^60^ ^]^ All three alkyl dicarbonates were detected for each of the cells using LP57 and account for ≈0.7 wt.% of the electrolyte in the case of DMDOHC and 1.5 wt.% for EMDOHC and DEDOHC, respectively.

**Figure 6:**
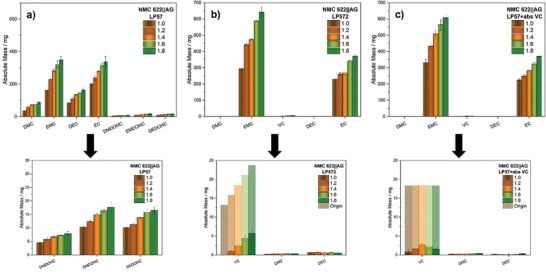
Determined absolute amounts of volatile electrolyte components of the NMC622||AG small‐format pouch cells (200 mAh) with LP57 (a), LP572 (b), and LP57 + absVC (c) at various vfs (1.0–1.8) after electrochemical aging. Low‐concentrated components are displayed enlarged with original VC amounts additionally presented transparently. Quantification was performed by means of GC‐FID.

Thus, considering the strong decrease in EMC and the formation of the observed aging products at considerable scales, a severely degradation of the electrolyte after 300 cycles can be concluded in terms of all cells. In the context of cell performance, this can result in irreversible capacity loss due to the reductive decomposition of the electrolyte components, as well as an increase in cell impedance due to thicker interphases or changes in essential physical properties of the electrolyte.^[^ ^19^, ^39^, ^60^ ^]^ However, the latter cannot dictate the differences occurring between the cell performance of the respective vfs (see Figure 2a), as there are no notable differences observed within the relative composition of the electrolyte after aging. Only the mass fractions of the linear carbonates of the cells with vf 1.0 exhibit a comparably slight decrease, indicating the onset of cell dryness due to electrolyte consumption. Due to the similar relative compositions of the electrolytes at each vf, a similar rate of the electrolyte decomposition by electrochemical reduction as well as subsequent chemical reactions involving alkoxide species can be inferred. For higher electrolyte quantities, this consequently implies the formation of correspondingly larger absolute amounts of degradation products with increasing vf, as evident in **Figure** **6**a. While the electrochemical reduction is, however, limited to the same surface area of the negative electrode, the extent of the subsequent reactions triggered by reduction products and thus presumed different growth of the interphase layers is mainly responsible for the observed differences in cell performance.

**Figure 7:**
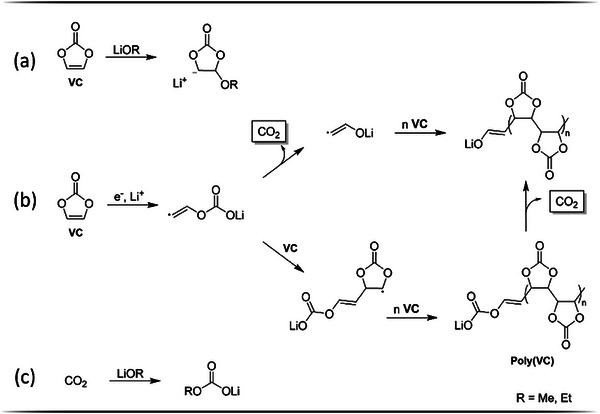
Reaction scheme for alkoxide scavenging by VC (a) according to Sasaki et al.,^[^ ^62^ ^]^ VC reduction with subsequent polymerization (b) according to Zhang et al.^[^ ^63^ ^]^ and alkoxide scavenging by CO_2_ (c) according to Zhang et al.^[^ ^20^ ^]^

As previously mentioned in Section 3.2, VC can be used to inhibit excessive decomposition of the electrolyte. Figure 4b shows the determined mass fractions of the detected components for the cells with the established LP572 (2 wt.% VC) electrolyte after electrochemical aging. The increasing total amounts of VC within the cell system with increasing vf due to the relative concentration of 2 wt.% becomes particularly evident by comparison of the remaining additive portions. While for cells with vf 1.0 the VC has been completely consumed during operation, the remaining portion of additive stepwise increases up to 0.47 wt.% (vf 1.8) with increasing vf. Nevertheless, compared to the additive‐free electrolyte, the positive effect of VC inhibiting excessive solvent decomposition is evident for each vf, as the previously observed reduction in linear carbonate is primarily minimized. Consequently, the transesterification products DMC and DEC are only present in minor proportions to an average of 0.03 and 0.06 wt.%, while the alkyl dicarbonates remain below the limit of detection. However, in terms of vf 1.0, where VC is entirely consumed, a more pronounced decrease in EMC is already evident, indicating the onset of solvent degradation processes. This particular dependence on VC availability is already well reflected in the formation of DEC, where the albeit low generated portions decrease with the absolute additive amount (Figure 4b). In literature, the protection against extensive electrolyte decomposition is usually attributed to the formation of an effective SEI due to the preferential reduction of VC. The nature of the SEI is described to be predominantly determined by poly(VC), which is formed after initial reduction of VC via radical polymerization.^[^ ^42^, ^44^, ^61^ ^]^ However, the organic polymer layer alone cannot protect the electrolyte from decomposition. Since the transference number of the SEI for unsolvated lithium‐ions is less than 1, solvated lithium cations and other electrolyte components can partially be transported through the interphase layer.^[^ ^10^ ^]^ Therefore, it was further found that despite the presence of VC, simultaneous EC reduction occurs within the early cycles, which was accordingly attributed to the dominance of EC in the solvation shell of the lithium cation.^[^ ^53^ ^]^ The alkoxide species generated during reduction of EC can eventually promote the degradation process of the electrolyte through surface independent chemical reactions.^[^ ^48^ ^]^ While the results suggest the presence of alkoxides by the formation of DMC and DEC, the extent of the decomposition is observed to be relatively low. In literature, this contradiction is addressed by the assumption of a scavenging effect of alkoxide species through VC. According to Sasaki et al.,^[^ ^62^ ^]^ VC is vulnerable to a nucleophilic attack, wherein alkoxide species are trapped by forming alkoxy‐EC (**Figure** **7**a). Alternatively, the reduction of VC results in the formation of radical anions accompanied by the release of CO_2_ (Figure 7b).^[^ ^52^, ^63^ ^]^ While the radical intermediates can form poly(VC) through chemical reaction with VC, Zhang et al.^[^ ^20^ ^]^ proposed a scavenging of the alkoxide species by CO_2_ (Figure 7c).

Since the results show that the reduction of EC and EMC were inhibited and subsequent degradation processes were almost completely suppressed, the differences in cell performance between the vfs can be attributed, as previously assumed, to the variation of VC quantities in the electrolyte. A higher total amount of additive within the cell system thus enables a higher total consumption of VC, as evident from Figure 6b. While a total amount of 13.2 mg was consumed for the cells with vf 1.0. the consumption increases with the vf up to 17.9 mg for vf 1.8. This higher overall consumption implicates a pronounced polymerization of VC, resulting in the formation of a thicker SEI. As implied in Figure 3, this leads to an increase of cell impedance despite the exclusion of electrolyte aging phenomena, ultimately deteriorating the cell performance to different extents.

By providing the same absolute amounts of VC for each vf, this problem should be overcome. The electrochemical data show that the differences in cell performance between the respective vfs were notably minimized by utilizing the LP57+abs. VC electrolyte. Figure 4c provides the determined mass fractions of the electrolyte components recovered from these specific cells. Similar to the results of the LP572 electrolyte, the presence of VC exerts a beneficial effect, as evidenced by the absence of a notable loss of the two main components EC and EMC as well as the complete suppression of alkyl dicarbonate formation. In fact, a direct comparison of the cells with vf 1.0, moreover, reveals no indication of a pronounced EMC degradation, suggesting the onset of electrolyte degradation processes. Thus, only minor changes in the quantification of the carbonate solvents are observable between the vfs, which is also reflected in the formation rate of the transesterification products DMC and DEC. This can primarily be attributed to the identical amount of additive available at all vfs, ensuring an inventory of VC even after 300 cycles, especially for the cells vf 1.0 with the lowest electrolyte quantity.

Accordingly, a proportional decrease of additive across all vfs could be assumed. Instead, the highest consumptions and thus lowest mass fractions with 0.09 and 0.11 wt.% of VC were observed for both cells with vf 1.0 and vf 1.8, respectively. Overall, the remaining portions of VC after electrochemical aging exhibit a pyramidal pattern, wherein the shares decrease with increasing/decreasing electrolyte volumes, starting from the cells with vf 1.4 (0.30 wt.%). This controversial behavior might be attributed to two different processes.

Generally, the same absolute amount of VC in decreasing electrolyte volumes results in an increasing concentration and vice versa. Thus, a higher VC concentration leads to an increased abundance within the electrolyte medium, a higher additive availability at the electrode surface and, consequently, an accelerated reaction rate between the VC molecules. The VC consumption in terms of lower electrolyte volumes would therefore be dominated by the VC polymerization (Figure 7b). In contrast, lower VC concentrations might therefore allow an enhanced simultaneous degradation process of the carbonate solvents, alongside the reduction of VC. Since the quantitative data only show a minor rise in aging products, the increased occurrence of concurring reactions to the VC polymerization in terms of the alkoxide scavenging is reasonable as the dominating process of VC consumption at higher electrolyte volumes (Figure 7a). These assumptions would further support the observations made for the cell performance. While the cells with a lower vf (1.0–1.4) exhibit comparatively less initial capacity due to potentially higher impedances caused by thicker SEI layers (besides worse wetting degrees), a superior capacity retention is observed compared to the cells with vf 1.6 and 1.8. The deterioration in capacity retention for higher vfs, particularly within the first 25 cycles, might thus be linked to a slightly increased reductive decomposition of the carbonate solvents accompanied with the irreversible loss of lithium‐ions. To ultimately validate and understand the proposed shift in working mechanism, the change of the SEI composition could be studied by surface‐sensitive quantitative spectroscopic characterization techniques such as X‐ray photoelectron spectroscopy.

### 3.4 Electrode Analysis

The difference in capacity fading among the varying vfs and between the different electrolyte compositions were attributed to the extent of various aging mechanism, as demonstrated by the electrolyte analysis. The involvement of the electrode surface plays a crucial role in these occurring aging processes, whether through the reduction of the individual electrolyte components or the alterations of the interphase layers. Thus, ICP‐OES measurements were performed to quantify the lithium content in the electrodes, locate the lost lithium within the cell system and ultimately support the conclusions drawn from the preceded investigations.

While the capacity fading of LIBs generally is an indicator for the irreversible loss of active lithium, kinetic hinderance of lithium‐ion transport in the electrodes and at the interphases also results in reduced cell capacities.^[^ ^64^ ^]^ However, after relaxation of the system, e.g., by paused cycling or application of lower currents, the apparently lost capacity can be recovered (see Figure 1b).^[^ ^65^ ^]^ Thus, in order to determine the actual irreversible loss of active lithium, the cells were discharged with a CV step in the last cycle to maximize the removal of active lithium from the anode. However, ICP‐OES analysis only provides information about the total lithium content within the individual electrodes. The technique does not differentiate whether the lithium originates from the SEI, plated metallic lithium, intercalated lithium or conductive salt.^[^ ^66^ ^]^ To minimize the influence of conductive salt residues, the electrode samples were thoroughly washed prior to analysis.

**Figure 8:**
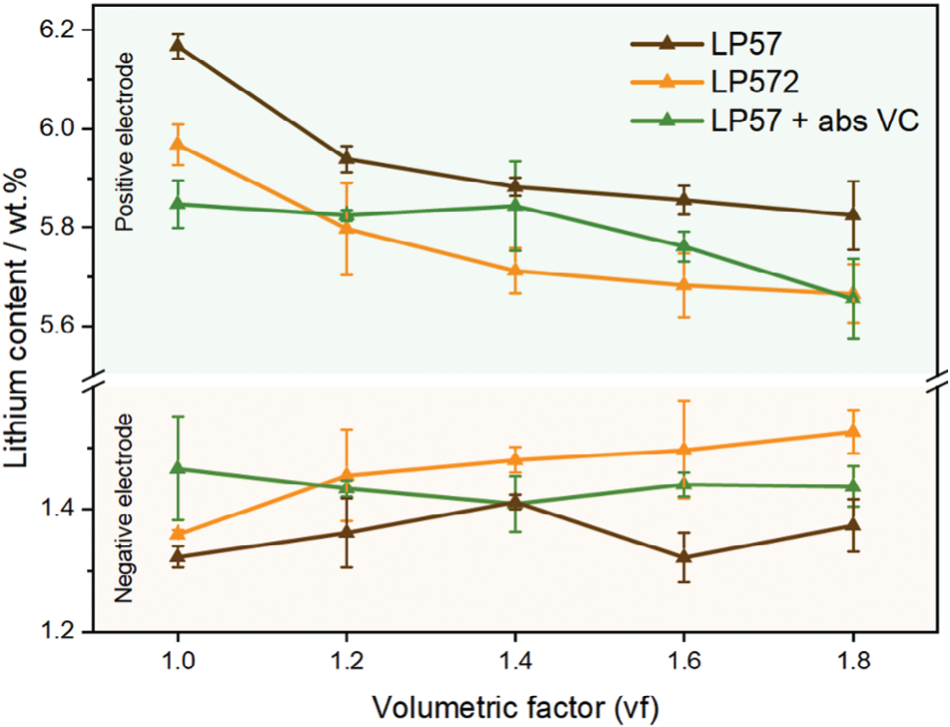
Quantified lithium contents in the active material of anode (bottom) and cathode (top) samples after electrochemical aging for 300 cycles plotted against the vf for the electrolytes LP57 (brown), LP572 (yellow), and LP57+absVC (green).

**Figure** **8** shows the lithium content by weight in the active material as determined *post mortem* by ICP‐OES. The results reveal a decreasing lithiation degree of the positive electrode with increasing vf for the LP57 and LP572 electrolytes. This trend is in good agreement with the capacity decay observed in the cycling data, as the capacity retention deteriorates with rising quantities of both electrolytes, eventually implying the identified decrease in the degree of lithiation.

Cells using LP57+absVC electrolyte show a different behavior. The data do not follow an immediate downward trend as for the two other electrolytes. Instead, the lithium content remains constant up to a vf of 1.4, before a further increase in electrolyte volume (vf >1.4) also leads to a decreasing trend in the determined values. This observation is also supported by the fact that the difference in cell performance is less pronounced when a constant amount of VC is used, leading to a similar additive consumption and thus presumably similar lithium contents. However, the decreasing lithium content for vf 1.6 and 1.8 might be explained through the slightly more severe capacity decay observed for these filling factors (compare Figure S1, Supporting Information). In addition, the proposed shift in working mechanism of VC due to slightly pronounced parasitic side reactions for higher vfs is supported by this trend.

On the negative electrode side, cells filled with LP572 show increasing lithium contents for higher vfs. Meanwhile, the lithium content for cells using LP57 + absVC and LP57 does not exhibit an explicit trend and remains on a constant level, regardless of the electrolyte filling volume. However, the latter is subject to considerably higher deviations among the various vfs.

These observations are in good agreement with the interpretations drawn from the electrolyte investigations. The quantification of the remaining VC content in the aged LP572 electrolyte demonstrated that more additive was consumed in cells filled at a higher vf due to the increased additive abundance. With the electrochemical initiation of the VC polymerization active lithium is consumed and incorporated into the SEI. Hence, the increase in lithium values with the vf can be explained by higher amounts of lithium being immobilized in the SEI due to the higher amount of VC per cell and more initialization reactions taking place.

For the LP57+absVC electrolyte, each cell contains the same amount of additive. The constant value for lithium measured among all vfs is therefore explicable by an equal amount of VC available for SEI formation. Even though slightly different amounts of VC were consumed, the majority of additive (>80 %) was absent for each cell after cycling. Hence, similar amounts of lithium immobilized in the interphase are expected.

Contrarily, the LP57 electrolyte contains no VC to be consumed. Accordingly, an enhanced formation of alkyl dicarbonates and transesterification products due to the absence of film‐forming additives was demonstrated. These parasitic side reactions were found to be more pronounced at higher vfs. Since lithium‐ions are irreversibly consumed during the initial reduction of the electrolyte components forming reactive alkoxide species, increasing loss of active lithium is therefore observable in the declining lithiation degree of the positive electrode. However, the lithium values in the negative electrode do not show the opposite trend and fluctuate around a constant level independently of the vf. Although active lithium is consumed forming lithium alkoxide species, the inorganic lithium salts are only partly incorporated in the SEI since the compounds catalytically take part in the chemical formation of the transesterification products, alkyl dicarbonates, and further oligomeric species. However, the formation and deposition of these aging products does not involve the immobilization of additional lithium‐ions. Observable fluctuations of the lithium content between the vfs might therefore be attributed to spatial heterogeneities of SEI growth or lithium plating which can lead to locally increased contents. This might be promoted due to the absence of film‐forming additive and the positive effects on the formation of homogeneous surface‐films.

To further support these conclusions and to elucidate the source of lithium deposition, the anode surface morphologies were investigated complimentarily to compare differences among the varying vfs and electrolytes used.

### 3.5 Morphology Investigations

**Figure 9:**
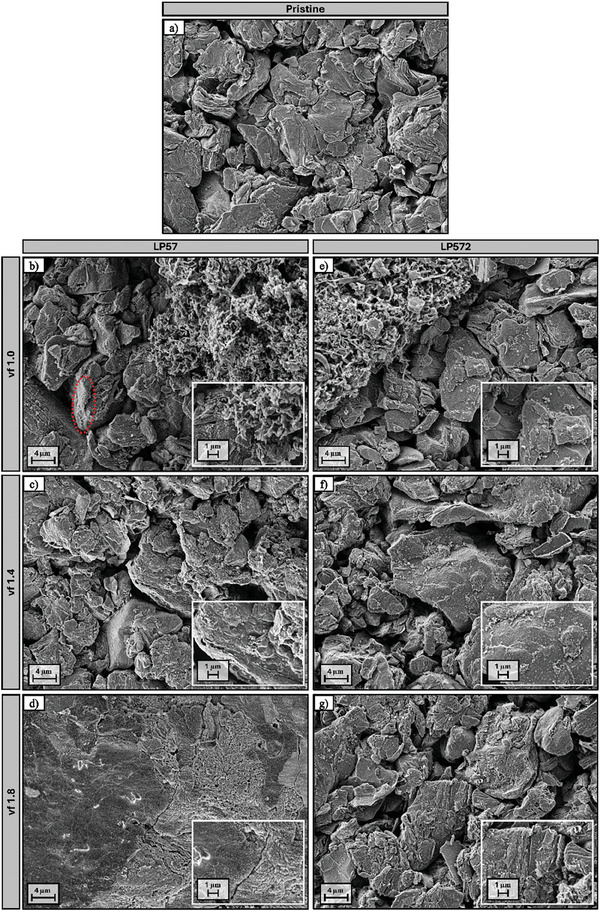
SEM investigations of the surface morphology of the pristine (a) electrochemically aged negative electrodes with LP57 at vf 1.0 (b), vf 1.4 (c), and vf 1.8 (d) as well as LP572 at vf 1.0 (e), vf 1.4 (f), and vf 1.8 (g). Smaller images show parts of a magnified area.

SEM was employed to visualize the morphological differences between the respective vfs of the negative electrodes in order to allow the preceding analytical investigations to be verified. The images of the pristine and cycled negative electrodes of cells filled with LP57 and LP572 at vfs of 1.0, 1.4, and 1.8 are shown in **Figure** **9**. Additionally, higher magnifications enable better insights of the particle surface conditions. Since SEM measurements are usually confined to a localized area of a large surface, emphasis was given to the selection of representative spots to reflect the overall impression of the cell.

The pristine electrode (Figure 9a) serves as a reference, showing the untreated flake‐like graphite particles with discernible layered basal planes. Smaller spherical particles between the graphite flakes can be assigned to the conductive agent. The cycled cells exhibit noticeable differences in morphology compared to the reference as well as amongst each other. These differences are evident not only in dependence on the electrolyte used but also based on the quantities of electrolyte.

After electrochemical aging with lower electrolyte volumes at vf 1.0, the formation of high surface area lithium (HSAL) was observed at various spots across the electrode.^[^ ^45^ ^]^ The formation and growth of HSAL is the result of heterogenous Li deposition and dissolution during battery operation and can be induced by various factors such as low temperatures, high C‐rates or inhomogeneous currents, and potential distributions.^[^ ^10^, ^45^ ^]^

Figure 9b shows the surface of the electrochemically aged negative electrode cycled with the additive‐free electrolyte LP57 at the respective low vf of 1.0. The section of the surface shows a boundary region of graphite particles and formed HSAL. The deposited lithium appears in an often described mossy‐like structure.^[^ ^67^, ^68^, ^69^ ^]^ Cells filled with low volumes are particularly susceptible to insufficient wetting and drying out due to electrolyte consumption during continuous operation, as previously shown in Figures 1 and 3. Accordingly, this can result in both non‐uniform current densities and an increase in internal resistance, thereby promoting the observed occurrence of lithium plating.^[^ ^70^, ^71^, ^72^ ^]^ However, since the lithium deposits are non‐specifically distributed in form of small clusters across the entire electrode, the contributing lithium content does not impact the preceding ICP‐OES measurements. Further, the graphite particles also exhibit morphological changes after electrochemical aging. While the original flake shape remains discernible, the particles feature surface depositions in form of the SEI. The magnification indicates a thin and more uniform interphase formed on the basal plane of the graphite, in contrast to the noticeably thicker appearing layer on the edge plane (marked, red oval). This observation is consistent with literature, in which higher reaction currents are perceived at the edge plane and a correspondingly several nm thicker SEI compared to the basal plane is assumed.^[^ ^15^, ^73^ ^]^

Increasing the LP57 electrolyte volume to a vf of 1.4 further amplifies these observations (Figure 9c). Even though lithium plating was absent based on presumably better wetting conditions, the particles exhibit an incrementally rougher and less uniform surface due to the increase of deposited SEI species on the basal and edge planes. Hence, the filling with increasing amounts of excess electrolyte accordingly leads to more visible deposition on the electrode surface. This finding is consistent with the results obtained from the GC measurements (Figure 6a), which revealed higher absolute amounts of aging products with rising LP57 electrolyte volumes. In case of vf 1.8, Figure 9d exposes this extensive electrolyte decomposition for large areas across the electrode surface, covering the graphite particles to be no longer discernible. At these areas, the SEI exhibits such thickness and rigidity to display cracks at multiple positions, allowing progressive electrolyte decomposition in terms of freshly formed SEI. In addition to the described rough areas, the electrode also features spots with a smoother surface, as depicted in Figure 9d (left). To investigate the differences in composition and the origin of the two surface morphologies, EDX was employed using a windowless detector enabling the detection of lithium. The composition of the acquired point spectra (Figure S3, Supporting Information) thus clearly identifies the rough surface layer as SEI, based on the element distribution of O/P/F/C (Table S5, Supporting Information, Spectrum 1), while the smoother surface can be attributed to the deposition of metallic lithium (Table S7, Supporting Information, Spectrum 3). Hence, the lithium plating demonstrates how excessive electrolyte decomposition can impede the lithium‐ion diffusion into the graphene layers, deteriorating the performance and safety of the cells.

Figure 9e–g shows the SEM images of the negative electrodes for the cells with LP572 at vf 1.0, 1.4, and 1.8. As for the cells with LP57, the cells with a low vf of 1.0 (Figure 9e) exhibit small spots of deposited lithium in form of a mossy structure. However, the graphite particles possess a thinner and more uniform appearing SEI when VC is used, which is particularly noticeable on the edge planes. While also no sign of HSAL formation was observed as the electrolyte volume was increased to vf 1.4 (Figure 9f), in contrast to LP57, the electrode surface is not subject to a more pronounced deposition of SEI species. Instead, the graphite particles indicate no noteworthy differences in the formed interphases on either the basal or edge planes compared to vf 1.0. Alterations in particle morphology by increased deposition of SEI species start to become evident with larger amounts of excess electrolyte in case of vf 1.8 (Figure 9g). This finding is particularly apparent when comparing the magnified images of the three different vfs. Based on the collected GC data, the observed changes can be mainly attributed to the increased reduction and polymerization of VC during electrochemical operation. Thus, despite the adverse impact of the excessive VC consumption on the cell impedance, the protective function against electrolyte decomposition is still ensured compared to LP57. Furthermore, after the selected 300 cycles, the occurrence of lithium plating due to the thickening of the interphases is not evident to this point.

Overall, the morphological comparison demonstrates and confirms that both the change of electrolyte as well as the alteration of the utilized volume have various impacts on the aging behavior of the cell, ultimately reflected by the electrochemical performance.

## 4 Conclusion

This study aimed to identify the influence of varying electrolyte quantities (represented by the volumetric factor vf) on the responsible aging processes deteriorating the cell performance during battery operation. Investigations of large‐format pouch cells with LP572 electrolyte demonstrated the impact of electrolyte quantity on the long‐term cycling behavior. The electrochemical data indicated the existence of an optimal electrolyte volume for achieving peak performance using a distinct cell system. Insufficient electrolyte volumes inevitably lead to premature drying out of the cell due to the continuous electrolyte consumption during operation, whereas excessive volumes result in increasingly accelerated capacity fading. To investigate the underlying aging mechanisms in more detail, controlled electrochemical aging with small‐format pouch cells was performed, enabling subsequent *post mortem* analysis on three types of electrolytes: LP57, LP572 (2 wt.% VC), and LP57 with a constant absolute amount of VC (18.351 mg). Complementary analysis methods, namely GC‐MS/FID, ICP‐OES, SEM, and EDX, were applied to gain comprehensive information about responsible degradation processes regarding the electrolyte, interphase layers, and the electrode morphology.

For both LP57 and LP572, the increase of electrolyte volume leads to a noteworthy decrease in cell performance. The capacity fading with an increase in electrolyte quantity could be attributed to different aging mechanisms for both electrolytes, respectively. Cells utilizing LP57 were found to primarily suffer from overall electrolyte decomposition. While the reduction of electrolyte components is accompanied by irreversible loss of lithium‐ions, especially the subsequent chemical reactions triggered by alkoxide species lead to higher absolute amounts of degradation products with increased electrolyte volume. This was accordingly reflected by a thicker SEI at the negative electrode surface, resulting in both an increase in cell impedance and the occurrence of lithium plating.

In contrast, the use of LP572 with 2 wt.% VC enabled an effective protection at all vfs against excessive electrolyte decomposition. Nevertheless, the defined 2 wt.% contributes to a higher absolute amount of additive as the electrolyte volume increases. This inevitably leads to more pronounced consumption of VC through reduction and further polymerization, forming thicker interphases and thus a likewise rise in cell impedance.

However, the results further show that electrolyte volumes chosen too low provoke insufficient wetting of the electrodes, which favors the plating of lithium, while the continuous electrolyte consumption during long‐term cycling ultimately ends in a faster drying out and thus in the rollover failure of the cells.

By varying the electrolyte quantity while keeping the absolute amount VC constant within the cell system, the observed differences in decreasing cell performance were considerably minimized. This adaptation, especially at higher vfs, allowed to counteract both excessive electrolyte decomposition and the formation of unfavorably thick SEI by excessive VC consumption. However, it was further found that the available electrolyte medium in relation to the concentration of VC contained therein can have an impact in the consumption rate or working mechanism of the additive.

The results emphasize that the commonly employed approach of using a defined percentage share of additive in the electrolyte is inadequate. Instead, it is preferential to tailor the electrolyte scales to the cell properties such as the pore volume, electrode surface area and amount of active material. This includes both the concentration of the additive as well as the utilized electrolyte quantity itself. This factor is often overlooked in literature, particularly for the comparability of different cell studies. Accordingly, the tests and analytical techniques applied in this study can be considered as a blueprint to optimize and evaluate the desired electrolyte formulations with respect to the cell design and material system used.

## Conflict of Interest

The authors declare no conflict of interest.

## Author Contributions

C.‐T.L. performed conceptualization, methodology, validation, formal analysis, investigation writing – original draft, writing – review and editing, visualization, and project administration. J.B. performed conceptualization, methodology, validation, formal analysis, investigation, writing – original draft, writing ‐review and editing, and project administration. J.H. performed conceptualization, methodology, investigation, resources, writing – original draft, writing – review and editing, and project administration. M.M.B. performed investigation. S.v.W. performed conceptualization. S.S. performed investigation, writing – review and editing. R.D. performed writing – review and editing, supervision, and funding acquisition. S.W.‐M. performed writing – review and editing, supervision, and funding acquisition. M.W. performed writing – review and editing, supervision, and funding acquisition. S.N. performed writing – review and editing, supervision, and funding acquisition.

## Supporting information

Supporting Information

## Data Availability

Research data are not shared.

## References

[1] T. Watari , B. C. McLellan , D. Giurco , E. Dominish , E. Yamasue , K. Nansai , Resour., Conserv. Recycl. 2019, 148, 91.

[2] M. Yoshio , in Lithium Ion Batteries, Springer, New York 2009.

[3] A. Thielmann , A. Sauer , M. Wietschel , in Gesamt‐Roadmap Lithium‐Ionen‐Batterien 2030, Fraunhofer‐Institut für System‐ und Innovationsforschung, ISI, Karlsruhe, 2015.

[4] M. M. Thackeray , C. Wolverton , E. D. Isaacs , Energy Environ. Sci. 2012, 5, 7854.

[5] O. Gröger , H. A. Gasteiger , J.‐P. Suchsland , J. Electrochem. Soc. 2015, 162, A2605.

[6] A. Kwade , W. Haselrieder , R. Leithoff , A. Modlinger , F. Dietrich , K. Droeder , Nat. Energy 2018, 3, 290.

[7] H. Xiong , E. J. Dufek , L. K. Gering , in Comprehensive Energy Systems, Elsevier, Amsterdam, Netherlands 2018, pp. 629–662.

[8] D. L. Wood , J. Li , C. Daniel , J. Power Sources 2015, 275, 234.

[9] J. Hagemeister , S. Stock , M. Linke , M. Fischer , R. Drees , M. Kurrat , R. Daub , Energy Tech 2022, 11, 2200686.

[10] J. Vetter , P. Novák , M. R. Wagner , C. Veit , K.‐C. Möller , J. O. Besenhard , M. Winter , M. Wohlfahrt‐Mehrens , C. Vogler , A. Hammouche , J. Power Sources 2005, 147, 269.

[11] E. Peled , S. Menkin , J. Electrochem. Soc. 2017, 164, A1703.

[12] J. Zhao , Z. Lu , H. Wang , W. Liu , H.‐W. Lee , K. Yan , D. Zhuo , D. Lin , N. Liu , Y. Cui , J. Am. Chem. Soc. 2015, 137, 8372. 26091423 10.1021/jacs.5b04526

[13] M. Winter , Zeitschrift für Physikalische Chemie 2009, 223, 1395.

[14] V. Agubra , J. Fergus , Materials 2013, 6, 1310. 28809211 10.3390/ma6041310 PMC5452304

[15] S. J. An , J. Li , C. Daniel , D. Mohanty , S. Nagpure , D. L. Wood , Carbon 2016, 105, 52.

[16] F. J. Günter , C. Burgstaller , F. Konwitschny , G. Reinhart , J. Electrochem. Soc. 2019, 166, A1709.

[17] X. Lai , Y. Li , R. Fang , P. Dong , Y. Zheng , Z. Li , J. Energy Storage 2022, 52, 104951.

[18] R. Fang , P. Dong , H. Ge , J. Fu , Z. Li , J. Zhang , J. Energy Storage 2021, 42, 103013.

[19] X. Han , L. Lu , Y. Zheng , X. Feng , Z. Li , J. Li , M. Ouyang , eTransportation 2019, 1, 100005.

[20] Sheng Shui Z. , J. Power Sources 2006, 162, 1379.

[21] Y. Qian , C. Schultz , P. Niehoff , T. Schwieters , S. Nowak , F. M. Schappacher , M. Winter , J. Power Sources 2016, 332, 60.

[22] S. Solchenbach , D. Pritzl , E. J. Y. Kong , J. Landesfeind , H. A. Gasteiger , J. Electrochem. Soc. 2016, 163, A2265.

[23] S. J. An , J. Li , D. Mohanty , C. Daniel , B. J. Polzin , J. R. Croy , S. E. Trask , D. L. Wood , J. Electrochem. Soc. 2017, 164, A1195.

[24] S. J. An , J. Li , C. Daniel , H. M. Meyer , S. E. Trask , B. J. Polzin , D. L. Wood , ACS Appl. Mater. Interfaces 2017, 9, 18799. 28505406 10.1021/acsami.7b03617

[25] S. Klick , G. Stahl , D. U. Sauer , Energy Tech 2024, 12, 2300566.

[26] B. R. Long , S. G. Rinaldo , K. G. Gallagher , D. W. Dees , S. E. Trask , B. J. Polzin , A. N. Jansen , D. P. Abraham , I. Bloom , J. Bareño , J. R. Croy , J. Electrochem. Soc. 2016, 163, A2999.

[27] D. Schreiner , T. Zünd , F. J. Günter , L. Kraft , B. Stumper , F. Linsenmann , M. Schüßler , R. Wilhelm , A. Jossen , G. Reinhart , J. Electrochem. Soc. 2021, 168, 030507.

[28] L. Terborg , S. Weber , S. Passerini , M. Winter , U. Karst , S. Nowak , J. Power Sources 2014, 245, 836.

[29] M. Grützke , X. Mönnighoff , F. Horsthemke , V. Kraft , M. Winter , S. Nowak , RSC Adv. 2015, 5, 43209.

[30] B. Vortmann‐Westhoven , M. Winter , S. Nowak , J. Power Sources 2017, 346, 63.

[31] M. Evertz , J. Kasnatscheew , M. Winter , S. Nowak , Anal. Bioanal. Chem. 2019, 411, 277. 30374724 10.1007/s00216-018-1441-8

[32] M. M. Bela , C. Schmidt , K. Neuhaus , T. Hering , M. C. Stan , M. Winter , M. Börner , Adv. Mater. Interfaces 2024, 11, 2300836.

[33] J. Groenewald , T. Grandjean , J. Marco , Renewable Sustainable Energy Rev. 2017, 69, 98.

[34] L. Ahmadi , A. Yip , M. Fowler , S. B. Young , R. A. Fraser , Sustainable Energy Technol. Assessments 2014, 6, 64.

[35] S. Klein , P. Bärmann , L. Stolz , K. Borzutzki , J.‐P. Schmiegel , M. Börner , M. Winter , T. Placke , J. Kasnatscheew , ACS Appl. Mater. Interfaces 2021, 13, 57241. 34813694 10.1021/acsami.1c17408

[36] F. J. Günter , S. Rössler , M. Schulz , W. Braunwarth , R. Gilles , G. Reinhart , Energy Tech 2020, 8, 1801108.

[37] T. Knoche , V. Zinth , M. Schulz , J. Schnell , R. Gilles , G. Reinhart , J. Power Sources 2016, 331, 267.

[38] J. S. Edge , S. O'Kane , R. Prosser , N. D. Kirkaldy , A. N. Patel , A. Hales , A. Ghosh , W. Ai , J. Chen , J. Yang , S. Li , M.‐C. Pang , L. Bravo Diaz , A. Tomaszewska , M. W. Marzook , K. N. Radhakrishnan , H. Wang , Y. Patel , B. Wu , G. J. Offer , Phys. Chem. Chem. Phys. 2021, 23, 8200. 33875989 10.1039/d1cp00359c

[39] Y. Liao , H. Zhang , Y. Peng , Y. Hu , J. Liang , Z. Gong , Y. Wei , Y. Yang , Adv. Energy Mater. 2024, 14, 2304295.

[40] K. Xu , Chem. Rev. 2014, 114, 11503. 25351820 10.1021/cr500003w

[41] D. Aurbach , K. Gamolsky , B. Markovsky , Y. Gofer , M. Schmidt , U. Heider , Electrochim. Acta 2002, 47, 1423.

[42] L. El Ouatani , R. Dedryvère , C. Siret , P. Biensan , S. Reynaud , P. Iratçabal , D. Gonbeau , J. Electrochem. Soc. 2008, 156, A103.

[43] J. C. Burns , R. Petibon , K. J. Nelson , N. N. Sinha , A. Kassam , B. M. Way , J. R. Dahn , J. Electrochem. Soc. 2013, 160, A1668.

[44] D. Pritzl , S. Solchenbach , M. Wetjen , H. A. Gasteiger , J. Electrochem. Soc. 2017, 164, A2625.

[45] G. Bieker , M. Winter , P. Bieker , Phys. Chem. Chem. Phys. 2015, 17, 8670. 25735488 10.1039/c4cp05865h

[46] T. Dagger , J. Kasnatscheew , B. Vortmann‐Westhoven , T. Schwieters , S. Nowak , M. Winter , F. M. Schappacher , J. Power Sources 2018, 396, 519.

[47] M. Lewerenz , G. Fuchs , L. Becker , D. U. Sauer , J. Energy Storage 2018, 18, 149.

[48] G. Gachot , S. Grugeon , M. Armand , S. Pilard , P. Guenot , J.‐M. Tarascon , S. Laruelle , J. Power Sources 2008, 178, 409.

[49] H.‐J. Peng , C. Villevieille , S. Trabesinger , H. Wolf , K. Leitner , P. Novák , J. Power Sources 2016, 335, 91.

[50] E. S. Takeuchi , H. Gan , M. Palazzo , R. A. Leising , S. M. Davis , J. Electrochem. Soc. 1997, 144, 1944.

[51] H. Yoshida , T. Fukunaga , T. Hazama , M. Terasaki , M. Mizutani , M. Yamachi , J. Power Sources 1997, 68, 311.

[52] B. Strehle , S. Solchenbach , M. Metzger , K. U. Schwenke , H. A. Gasteiger , J. Electrochem. Soc. 2017, 164, A2513.

[53] R. Stockhausen , L. Gehrlein , M. Müller , T. Bergfeldt , A. Hofmann , F. J. Müller , J. Maibach , H. Ehrenberg , A. Smith , J. Power Sources 2022, 543, 231842.

[54] D. Aurbach , Y. Ein‐Eli , B. Markovsky , A. Zaban , S. Luski , Y. Carmeli , H. Yamin , J. Electrochem. Soc. 1995, 142, 2882.

[55] F. Horsthemke , V. Winkler , M. Diehl , M. Winter , S. Nowak , Energy Tech 2020, 8, 1801081.

[56] J.‐S. Shin , C.‐H. Han , U.‐H. Jung , S.‐I. Lee , H.‐J. Kim , K. Kim , J. Power Sources 2002, 109, 47.

[57] T. Sasaki , T. Abe , Y. Iriyama , M. Inaba , Z. Ogumi , J. Power Sources 2005, 150, 208.

[58] C. Fang , T.‐N. Tran , Y. Zhao , G. Liu , Electrochim. Acta 2021, 399, 139362.

[59] J. Henschel , F. Horsthemke , Y. P. Stenzel , M. Evertz , S. Girod , C. Lürenbaum , K. Kösters , S. Wiemers‐Meyer , M. Winter , S. Nowak , J. Power Sources 2020, 447, 227370.

[60] T. Sasaki , S.‐K. Jeong , T. Abe , Y. Iriyama , M. Inaba , Z. Ogumi , J. Electrochem. Soc. 2005, 152, A1963.

[61] S. Grugeon , P. Jankowski , D. Cailleu , C. Forestier , L. Sannier , M. Armand , P. Johansson , S. Laruelle , J. Power Sources 2019, 427, 77.

[62] T. Sasaki , T. Abe , Y. Iriyama , M. Inaba , Z. Ogumi , J. Electrochem. Soc. 2005, 152, A2046.

[63] B. Zhang , M. Metzger , S. Solchenbach , M. Payne , S. Meini , H. A. Gasteiger , A. Garsuch , B. L. Lucht , J. Phys. Chem. C 2015, 119, 11337.

[64] T. R. Jow , S. A. Delp , J. L. Allen , J.‐P. Jones , M. C. Smart , J. Electrochem. Soc. 2018, 165, A361.

[65] B. Epding , B. Rumberg , H. Jahnke , I. Stradtmann , A. Kwade , J. Energy Storage 2019, 22, 249.

[66] P. Münster , M. Diehl , J. E. Frerichs , M. Börner , M. R. Hansen , M. Winter , P. Niehoff , J. Power Sources 2021, 484, 229306.

[67] L. Gireaud , S. Grugeon , S. Laruelle , B. Yrieix , J.‐M. Tarascon , Electrochem. Commun. 2006, 8, 1639.

[68] J. Yamaki , S. Tobishima , K. Hayashi , K. Saito , Y. Nemoto , M. Arakawa , J. Power Sources 1998, 74, 219.

[69] Z. Li , J. Huang , B. Y. Liaw , V. Metzler , J. Zhang , J. Power Sources 2014, 254, 168.

[70] R. Korthauer , in Lithium‐Ion Batteries: Basics and Applications, Springer, Berlin, Heidelberg 2018.

[71] N. Kaden , R. Schlimbach , Á. R. García , K. Dröder , Batteries 2023, 9, 164.

[72] D. H. Jeon , Energy Storage Mater. 2019, 18, 139.

[73] S. Tsubouchi , Y. Domi , T. Doi , M. Ochida , H. Nakagawa , T. Yamanaka , T. Abe , Z. Ogumi , J. Electrochem. Soc. 2012, 159, A1786.

