# Supplementary material for: Analyzing the Effect of Electrolyte Quantity on the Aging of Lithium‐Ion Batteries
Source: Adv Sci (Weinh). 2024 Aug 19;11(39):2405897. doi: 10.1002/advs.202405897 (PMC11497071; doi:10.1002/advs.202405897)
Supplement: Supplementary file 1 — Supporting Information [file ADVS-11-2405897-s001.pdf]

ADVANCED SCIENCE Open Access

Supporting Information

for *Adv. Sci.*, DOI 10.1002/advs.202405897

Analyzing the Effect of Electrolyte Quantity on the Aging of Lithium-Ion Batteries

*Christian-Timo Lechtenfeld, Julius Buchmann, Jan Hagemeister, Marlena M. Bela, Stefan van Wickeren, Sandro Stock, Rüdiger Daub, Simon Wiemers-Meyer, Martin Winter and Sascha Nowak**

Analyzing the Effect of Electrolyte Quantity on the Aging of Lithium-Ion Batteries

Christian-Timo Lechtenfeld[a], Julius Buchmann[a], Jan Hagemeister[b], Marlena M. Bela[a], Stefan van Wickeren[a], Sandro Stock[b], Rüdiger Daub[b], Simon Wiemers-Meyer[a], Martin Winter[a,c] and Sascha Nowak[a,*]

[a]*University of Münster, Münster Electrochemical Energy Technology (MEET), Corrensstraße 46, 48149 Münster, Germany*

[b]*Technical University of Munich (TUM), Institute for Machine Tools and Industrial Management (iwb), Boltzmannstr. 15, 85748 Garching, Germany*

[c]*Helmholtz-Institute Münster (HIMS), IEK-12, Forschungszentrum Jülich, Corrensstraße 46, 48149 Münster, Germany*

[*]*Corresponding author. E-Mail address: Sascha.nowak@uni-muenster.de*

Table S1. Filled electrolyte volumes of the large-format pouch cells represented by volumetric factors (vf).

vf	Electrolyte volume in mL
1.01	10.50
1.07	11.16
1.13	11.74
1.21	12.56
1.30	13.47
1.37	14.21
1.50	15.54
1.58	16.45

Table S2. Overview of the small-format pouch cell component porosities obtained by MIP and measures taken from an opened unfilled cell. V_{total} was calculated from the data to determine the vfs for electrolyte filling.

	Anode	Cathode	Separator
Porosity in %	35.97 ± 0.47	31.60 ± 0.83	59.63 ± 1.22
Coating length in cm	35.5	34.1	45.8
Coating width in cm	2.8	2.6	3.0
Coating thickness in mm	0.735	0.543	0.2
Foil thickness in mm	0.10 (Cu)	0.14 (Al)	-
Calculated $V_{\mathbf{pore}}$ in cm^3	0.2583	0.1504	0.1639
$V_{pore,total}$ in cm^3		0.5725	

Table S3. List of electrolyte filling volume of small-format pouch cells according to vf and VC amount of the used electrolytes.

vf	Electrolyte volume / mL	VC amount		
		LP572	LP57	Abs. VC
1.0	0.573	2 wt.%	0 wt.%	2.80 wt.%; 18.351 mg
1.2	0.687			2.32 wt.%; 18.351 mg
1.4	0.802			2.03 wt.%; 18.351 mg
1.6	0.916			1.81 wt.%; 18.351 mg
1.8	1.031			1.55 wt.%; 18.351 mg

Table S4. List of parameters applied for the GC measurements.

GC parameter	GC-FID<br>Shimadzu Nexis 2030	GC-MS<br>Shimadzu Nexis 2030
Carrier gas	Helium, 1.16 L min^{-1}	Helium, 1.0 L min^{-1}
Injection volume	1 µL	1 µL
Injection split	1:20	1:20
Injector temperature	250 °C	230°C
Temperature program	40 °C, 1 min<br>→ 80 °C, 12 K min^{-1}<br>→ 210 °C, 33 K min^{-1}	40 °C, 3 min<br>→ 60 °C, 3 K min^{-1}<br>→ 210 °C, 33 K min^{-1}

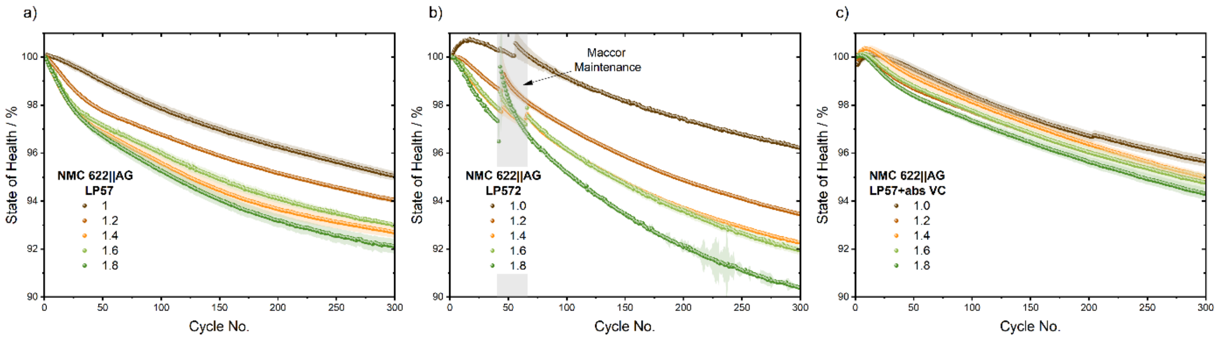

Figure S1. Normalized discharge capacities of NMC622||AG small-format pouch cells (200 mAh) with LP57 (a), LP572 (b) and LP57+absVC (c) electrolyte at different vfs varying from 1.0 to 1.8 (0.2 steps). Electrochemical cycling was conducted at 1C for 300 cycles in the voltage range of 2.9 V- 4.2 V.

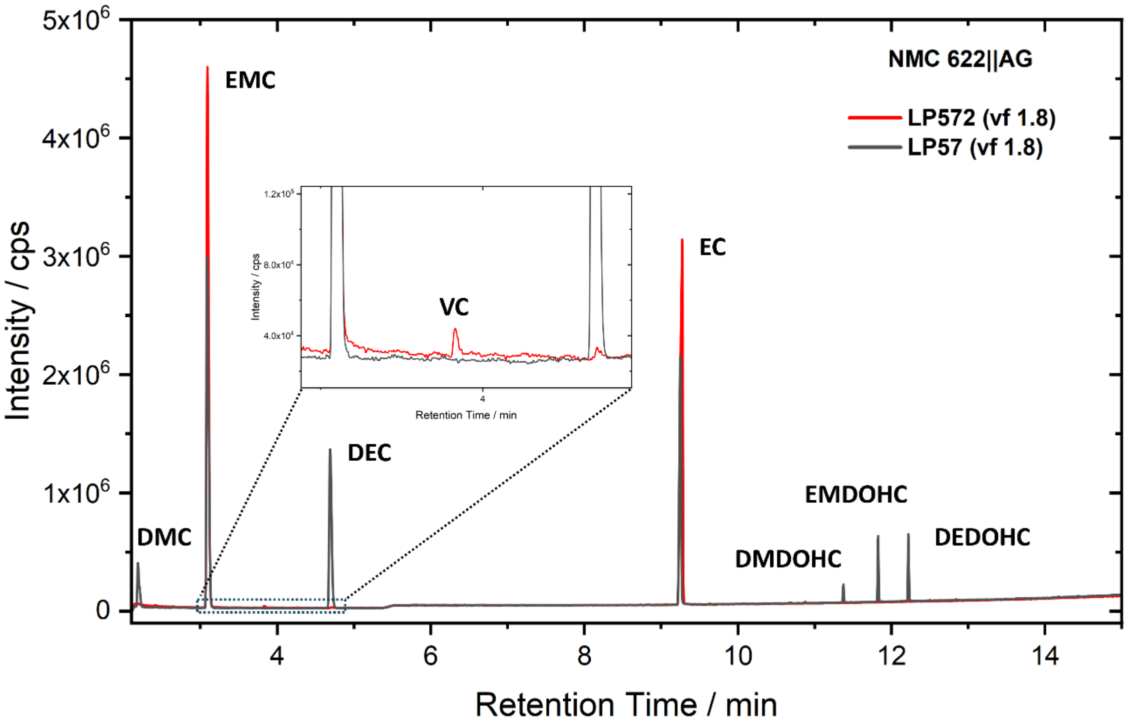

Figure S2. GC-MS chromatogram of identified electrolyte components for the electrochemically aged electrolytes from small-format pouch cells with the electrolytes LP57 and LP572 at vf 1.8.

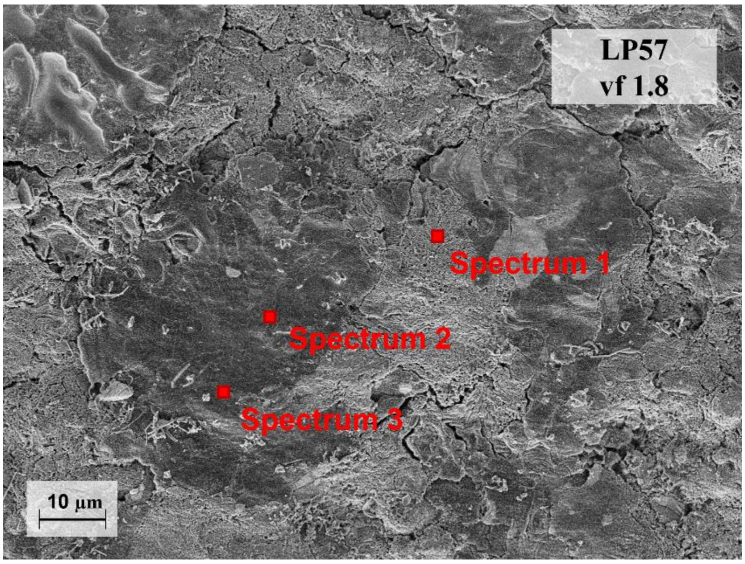

Figure S3. SEM image of the surface of the negative electrode electrochemically aged with LP57 at vf 1.8. The surface was used to acquire point spectra *via* EDX spectroscopy in order to investigate the surface composition. Three spectra at different positions were acquired.

Table S5. Determined surface composition of spectrum 1 acquired from the negative electrode electrochemically aged with LP57 at vf 1.8.

Element	Line Type	Mass Fraction / wt.%	Standard Deviation / wt.%
Lithium (Li)	K series	0.00	28.16
Carbon (C)	K series	18.12	0.24
Nitrogen (N)	K series	0.00	0.09
Oxygen (O)	K series	23.06	0.19
Fluor (F)	K series	39.12	0.26
Sodium (Na)	K series	2.82	0.10
Phosphorus (P)	K series	16.88	0.30
Total		100.00	

Table S6. Determined surface composition of spectrum 2 acquired from the negative electrode electrochemically aged with LP57 at vf 1.8.

Element	Line Type	Mass Fraction / wt.%	Standard Deviation / wt.%
Lithium (Li)	K series	69.68	3.10
Carbon (C)	K series	11.11	1.15
Nitrogen (N)	K series	0.29	0.10
Oxygen (O)	K series	8.26	0.85
Fluor (F)	K series	6.20	0.64
Sodium (Na)	K series	0.62	0.10
Phosphorus (P)	K series	3.83	0.44
Total		100.00	

Table S7. Determined surface composition of spectrum 3 acquired from the negative electrode electrochemically aged with LP57 at vf 1.8.

Element	Line Type	Mass Fraction / wt.%	Standard Deviation / wt.%
Lithium (Li)	K series	88.70	0.58
Carbon (C)	K series	5.96	0.34
Nitrogen (N)	K series	0.00	0.07
Oxygen (O)	K series	2.67	0.15
Fluor (F)	K series	1.47	0.10
Sodium (Na)	K series	0.57	0.06
Phosphorus (P)	K series	0.62	0.12
Total		100.00	
